# Extracellular Events Involved in Cancer Cell–Cell Fusion

**DOI:** 10.3390/ijms232416071

**Published:** 2022-12-16

**Authors:** Thomas Dittmar, Ralf Hass

**Affiliations:** 1 Institute of Immunology, Center for Biomedical Education and Research (ZBAF), Witten/Herdecke University, Stockumer Str. 10, 58448 Witten, Germany; 2 Biochemistry and Tumor Biology Laboratory, Department of Obstetrics and Gynecology, Hannover Medical School, 30625 Hannover, Germany

**Keywords:** cancer cell fusion, fusogens, post-hybrid selection process (PHSP)

## Abstract

Fusion among different cell populations represents a rare process that is mediated by both intrinsic and extracellular events. Cellular hybrid formation is relayed by orchestrating tightly regulated signaling pathways that can involve both normal and neoplastic cells. Certain important cell merger processes are often required during distinct organismal and tissue development, including placenta and skeletal muscle. In a neoplastic environment, however, cancer cell fusion can generate new cancer hybrid cells. Following survival during a subsequent post-hybrid selection process (PHSP), the new cancer hybrid cells express different tumorigenic properties. These can include elevated proliferative capacity, increased metastatic potential, resistance to certain therapeutic compounds, and formation of cancer stem-like cells, all of which characterize significantly enhanced tumor plasticity. However, many parts within this multi-step cascade are still poorly understood. Aside from intrinsic factors, cell fusion is particularly affected by extracellular conditions, including an inflammatory microenvironment, viruses, pH and ionic stress, hypoxia, and exosome signaling. Accordingly, the present review article will primarily highlight the influence of extracellular events that contribute to cell fusion in normal and tumorigenic tissues.

## 1. Introduction

Fusion processes are understood as a merger of phospholipid bilayer membranes of, e.g., vesicles (e.g., endocytosis and exocytosis), cell organelles (e.g., mitochondria, exosomes), infection of host cells with enveloped viruses, and hybridization of cells. The fusion process itself is a complex, energy-dependent, and tightly and temporally regulated event but only partially understood to date (for review, see: [1,2,3,4,5,6,7,8,9,10,11,12,13,14]). Cell fusion can occur as homofusion (fusion of the same cell type) or heterofusion (fusion between different cell populations) in the course of physiological developments, e.g., during formation of myocytes, osteoclasts, or syncytiotrophoblasts, among others [15]. Pathophysiological heterofusion, such as fusion of breast cancer cells with mesenchymal stroma-/stem-like cells (MSC), has been documented as a rapid event that occurs within a few minutes [16]. The resulting cancer hybrid cells enter a post-hybrid selection process (PHSP). Such PHSP is required to eliminate DNA-unstable karyotypes generated by chromosomal recombination of different nuclei during the previous cell merger [3,17,18]. Cancer hybrid cells have been identified in various tumor types, including breast, ovarian, gastric, and lung tumors following interaction and subsequent heterofusion of corresponding cancer cells with MSC [19,20,21,22]. Thus, cancer cell fusions may elevate the risk to develop new cancer stem-like cells [18,23]. The cancer cell × MSC fusion is predominantly related to an activated presence and the supportive functionality of MSC within the tumor microenvironment (TME). In general, MSC exhibit regenerative and repair activities in damaged tissues and are, therefore, also recruited to tissue lesions of invasively growing tumors [24,25,26,27]. At tumor sites, MSC also display their regenerative capacities, which simultaneously can support tumor growth. Fusion of cancer cells with MSC generates a variety of different hybrid subtypes with altered properties, including enhanced tumor growth and metastatic spreading [17], reduced tumorigenicity [22], or tumor dormancy [28]. Accordingly, cancer cell heterofusion contributes to significantly elevated tumor heterogeneity and plasticity [2,29,30].

### 1.1. Brief Facts of the Cell–Cell Fusion Machinery

In recent years, a number of proteins and phospholipids have been identified that play a crucial role in cell–cell fusion. These include so-called fusogens, such as syncytin-1 (human endogenous retrovirus-type W (HERV-W), syncytin-2 (human endogenous retrovirus-type FRD (HERV-FRD), Izimo1, Juno, myomaker, and myomerger. Fusogens catalyze fusion of two phospholipid double membranes that should actually repel each other due to their negative charge [1,2,3,4,5,6,7,8,9,10,11,12,13,14]. Similarly, phosphatidylserine (PS) is a phospholipid that has been proposed as a conserved “fuse me” signal regulating the time and place of cell–cell fusion and virus–cell fusion processes [6,10]. Appropriate receptors for syncytin-1 and PS must be expressed since their interactions are required for cell–cell fusion and virus–cell fusion, respectively. Neutralizing antibodies that interact with the receptor binding domain of the spike protein of severe acute respiratory syndrome coronavirus-2 (SARS-CoV-2) prevent binding to the ACE2 receptor and thus infection of the target cell [31]. Likewise, human endogenous retroviral (HERV) element suppressyn binds to syncytin-1 receptors alanine, serine, and cysteine transporter 2 (ASCT2) and thus prevents syncytin-1-mediated cell–cell fusion [32]. Furthermore, no multinucleated muscle cells were observed in myomaker–myomerger knockout embryos [33]. Similarly, infertility was correlated to either Juno (oocyte) or Izumo1 (sperm) knockout due to impaired sperm–oocyte fusion ability [34,35,36].

In addition to these key elements in cell–cell fusion, other proteins involved in this process have been identified, including cell adhesion molecules, F-actin and F-actin reorganizing proteins, proteases, chemokines, and cytokines [1,2,3,4,5,6,7,8,9,10,11,12,13,14,37]. F-actin and proteins involved in actin cytoskeleton reorganization, such as Arp2/3/WASP, Cdc42, and Rac1, have already been shown to be actively involved in cell–cell fusion of, e.g., myoblasts or breast cancer cells [8,37,38]. In this process, F-actin-based protrusions penetrate the target cells and cause fusion pores so that the cells fuse with each other. For the other protein classes mentioned, a direct connection with the fusion of the membranes has not yet been demonstrated. Nevertheless, chemokines and cell–cell adhesion molecules, for example, are essential for cell–cell fusion so that two cells can move toward each other and enter into contact [39].

For cell–cell fusion, it is assumed that cells have to acquire pro-fusogenic status in order to fuse [1,2,3,4,5,6,7,8,9,10,11,12,13,14]. In fact, cells are not fusogenic by themselves but must express certain proteins/factors, such as fusogens or PS, the appropriate receptors, to be able to fuse with each other. Similarly, once fusion has occurred, cells must revert to a non-fusogenic state to avoid undesirable subsequent fusion events. Cellular and molecular mechanisms that are important for expression of this hypothetical state remain to be explored. Likewise, clear definitions of pro-fusogenic state need to be articulated.

### 1.2. Physiological and Non-Physiological Cell–Cell Fusion

**Figure 1:**
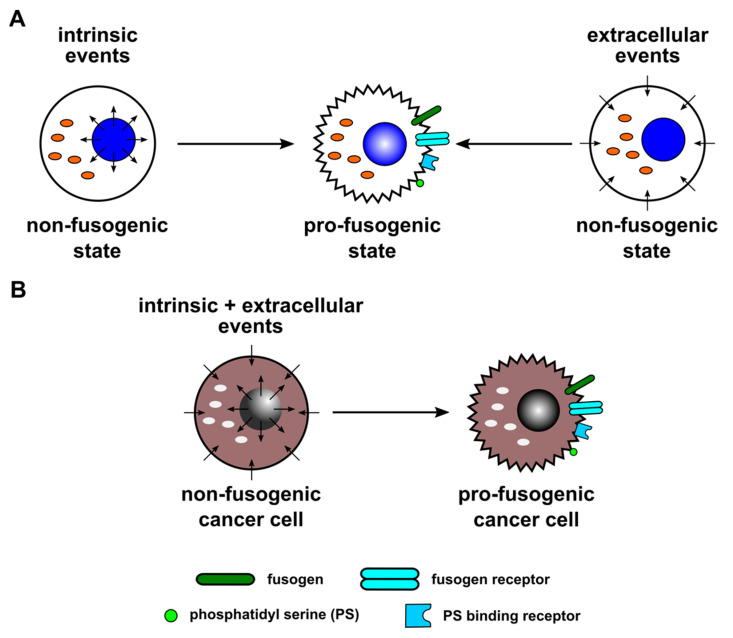
Both intrinsic and extracellular events can convert cells to a pro-fusogenic state, as postulated in this hypothetic model. (**A**) Intrinsic events likely play a role in physiological cell–cell-fusion-dependent processes, such as placentation and myogenesis. Here, intrinsic events lead to time-dependent and developmental-state-dependent expression of the major components of the cell–cell fusion machinery, which ensures a high frequency of cell–cell fusion events. In addition, extracellular events could also convert cells towards a pro-fusogenic state, which would enable BMDCs in tissue regeneration. In a normal state, BMDCs are non-fusogenic but could be converted into a pro-fusogenic state, which is most likely triggered by damaged tissue-derived extracellular signals. (**B**) Cancer cells could fuse either spontaneously or following induction, suggesting that both intrinsic and/or extracellular events are controlling this process.

Placental and muscle maturation represent physiological, genetically determined cell–cell fusion processes that are characterized by intrinsic expression of the major components of the cell–cell fusion machinery. These expression levels represent a pro-fusogenic state in a high number of cells, which ensures a high frequency of cell–cell fusion events to occur in parallel [1,2,3,4,5,6,7,8,9,10,11,12,13,14] (Figure 1A). In contrast, bone-marrow-derived cells (BMDCs) represent a population of cells that can regenerate tissue by cell–cell fusion but do not themselves possess intrinsic basal fusogenic properties [40,41,42,43]. While this regenerative capacity underlies regulatory controls, appropriate extracellular processes are also required to shuttle BMDCs into a pro-fusogenic state (Figure 1A).

In comparison, fusion of cancer cells is different since only a minor proportion of the cells exhibits fusogenic capacities. Given that about 1% of cancer cells can undergo homofusion or fuse with other cells, such as macrophages or MSC, this also means that 99% of cancer cells do not fuse. However, why are these 1% of cancer cells fusogenic, and how are they different as compared to the 99% non-fusogenic cancer cells? Several studies have shown that syncytin-1 is involved in cancer cell fusion [44,45,46,47]. However, the relative fusion rate is usually very low (mostly between 1% and 2%). This is in contrast to the high syncytin-1-mediated fusion frequency of cytotrophoblasts. Immunohistochemical studies of breast cancer, colorectal cancer, lung cancer, and endometrial cancer tissues revealed heterogeneous syncytin-1 expression among the cancer cells [45,46,48,49,50]. These data indicate that this fusogen is expressed only in a certain compartment of the tumor and/or in a specific subpopulation of cancer cells. In contrast, other studies revealed that syncytin-1 or SyHP were ubiquitously expressed in virtually all cancer cells [44,51]. Interestingly, all the studies had in common that syncytin-1 was mainly detectable in the cytosolic part and only partially in the cell membrane of the cancer cells [44,45,46,48,49,50,51]. This would be an explanation for the relatively low fusion rate of syncytin-1-expressing cancer cells. Only plasma-membrane-associated syncytin-1 is capable to mediate fusion.

### 1.3. Known Triggers for Cell–Cell Fusion

Triggers for induction of syncytin-1 translocation to the plasma membrane are represented by both irradiation and hypoxia. These extracellular conditions have been identified as cellular stress effectors also involved in formation of cell–cell fusion-derived polygiant cancer cells (PGCCs) [44,51]. Moreover, inflammation or inflammatory cytokines are known mediators that can induce cell fusion [16,46,52,53,54,55,56,57,58,59,60]. For example, fusion of BMDCs was approximately 10–100 times higher in chronic inflammatory tissue [53]. Similar observations occurred in the chronic inflammatory intestinal epithelium of mice [52]. In addition, it is known that fusion of cancer cells with macrophages or MSC can be induced by pro-inflammatory cytokine tumor necrosis factor-α (TNF-α) [16,46,55,56,57,58,61]. However, it remains unclear exactly how inflammatory processes or cytokines promote cell–cell fusion. Aside from induced expression of the fusogen syncytin-1 [46], TNF-α has also been described to induce expression of the matrix metalloproteinase-9 (MMP9) [56,58], which is important for cell–cell merger. Thus, inflammatory conditions and cytokine signaling may also contribute to plasma membrane translocation of syncytin-1.

While the process of cell–cell fusion is still not fully understood, some of the involved mechanistic processes have been identified [4,5,10]. However, knowledge regarding temporal orchestration of an appropriate molecular platform to control this process remains scarce. From a physiological point of view, this particularly applies to cells that do not constitutively provide any fusogenic properties but can nevertheless fuse with other cells, including cancer cells. The fact that cancer cells can fuse either spontaneously or following induction suggests the presence of intrinsic and/or extracellular factors controlling this process (Figure 1B). Intrinsic factors represent, e.g., proteins and phospholipids that are expressed by fusogenic cells themselves. This could be either constitutively or only at specific time points, such as syncytin-1 in cytotrophoblasts [62,63] or myomaker and myomerger in myoblasts [11,64], respectively. Likewise, extracellular factors represent events from the outside of cells to induce/mediate fusion processes. These events include, among others, inflammatory conditions and corresponding cytokines, hypoxia, low pH, viruses, and extracellular vesicles (EVs)/exosomes that will be discussed further in this review article.

## 2. Inflammation/Inflammatory Cytokines in Cell Fusion

Inflammation and inflammatory cytokines, such as TNF-α, are well-known triggers/inducers of cell–cell fusion that has been demonstrated in several studies [16,46,52,53,54,55,56,57,58,59,60]. Yan et al. demonstrated that TNF-α stimulation resulted in increased syncytin-1 expression levels in squamous cell carcinoma cells 9 (SCC-9) and its cognate receptor ASCT2 in human umbilical vein endothelial cells (HUVEC), which was correlated with increased fusion frequency of these cells [46]. These findings substantiate that fusogens and their corresponding receptors are mandatory for cell–cell fusion. Similarly, these data indicate that inflammation/inflammatory cytokines likely prime different cell types for subsequent plasma membrane fusion.

### 2.1. Role of TNF-α in Cell–Cell Fusion

Aside from this direct correlation, other studies showed a link between inflammation/inflammatory cytokines and cell fusion [52,53,54,55,60]. Other work reported that certain TNF-α-regulated proteins were involved in fusion [16,56,57,58,59]. For instance, Song and colleagues demonstrated that TNF-α could enhance oral squamous cell carcinoma (OSCC) × HUVEC fusion through the α_4_β_1_ (very late antigen-4 (VLA-4))/vascular cell adhesion molecule-1 (VCAM-1) pathway [59]. This is similar to α_4_β_1_ (VLA-4)/VCAM-1-dependent fusion of CD34^+^ blood cells and murine cardiomyocytes [65]. Both α_4_β_1_ (VLA-4) and VCAM-1 blocking antibodies markedly impaired TNF-α induced OSCC × HUVEC cell adhesion and cell–cell fusion [65]. However, this study only showed that TNF stimulation induced VACM1 expression in OSCC cells and HUVECs, whereas the effect on VLA-4 expression was not investigated [65]. In contrast, α_4_β_1_ (VLA-4) expression was up-regulated in TNF-α- and interleukin-6 (IL-6)-stimulated CD34^+^ blood cells, which was relevant for fusion with murine cardiomyocytes [65]. Fusion of human MSC and human MCF10A breast epithelial cells was significantly enhanced by TNF-α stimulation but impaired by siRNA-mediated knockdown of TNF receptor-1 and -2 and TRADD [16]. This substantiates the impact of TNF-α on cell–cell fusion. Interestingly, both syncytin-1 and syncytin-2 and its cognate receptors, ASCT-2 and MFSD2A, were expressed by MSC and MCF10A cells, but the fusion frequency of these cells remained unchanged after siRNA-mediated knockdown [16]. These data indicate that fusion of MSC and MCF10A cells is likely mediated by fusogens/proteins other than syncytin-1 and -2 and their receptors. 

### 2.2. Impact of Proteases and β-Catenin in Cell–Cell Fusion

TNF-α-induced expression of MMP-9 was involved in macrophage fusion and human M13SV1-EGFP-Neo breast epithelial cells × human MDA-MB-435 cancer cell hybridization [56,57,58,60]. Skokos et al. assumed that MMP-9 cleaves substrates, such as E-Cadherin, in which degradation might be necessary for cell–cell fusion [56]. Close cell–cell contact mediated by adhesion molecules is mandatory for cell–cell fusion. Hence, membrane proteins, including adhesion molecules, must be removed/digested to allow the plasma membranes of the two cells to approach each other. The cytoplasmic domain of E-Cadherin binds to β-catenin, which is linked to the cytoskeleton, thereby playing a role in regulation and coordination of cell–cell adhesion [66]. On the other hand, Wnt signaling via transcription co-activator β-catenin controls embryonic development and adult homeostasis [66]. Given that MMP-9 cleaves E-Cadherin in macrophages [56], it can be assumed that release of β-catenin to the cytoplasm could have an impact on cytoskeletal reorganization and an altered gene expression pattern, which might play a role in cell–cell fusion. Takezawa et al. reported that β-catenin might be a molecular switch that regulates transition of cell–cell adhesion to fusion [67]. Thereby, at fertilization, adhesion of sperm to the surface of an oocyte is mediated by the E-Cadherin/β-catenin complex [67]. Moreover, subsequent rapid proteasomal degradation of β-catenin in the oocytes is required for sperm × oocyte fusion [67]. These findings likely indicate that β-catenin-dependent sperm × oocyte fusion is rather attributed to reorganization of the cytoskeleton in the oocyte.

Maretzky and colleagues demonstrated that ADAM10-mediated cleavage of E-Cadherin was associated with β-catenin translocation from the plasma membrane to the cytoplasm and nucleus and expression of β-catenin downstream genes [68]. Similarly, ADAM12-directed ectodomain shedding of E-Cadherin potentiated trophoblast fusion, most likely through induction of glial cell missing-1 (GCM1) and syncytin-1 expression [69]. Indeed, studies of Matsuura et al. demonstrated that β-catenin/BCL-9-like (BCL9)/T-cell factor 4 (TCF4) signaling directly targeted the GCM1/syncytin pathway and thereby regulated fusion of human choriocarcinoma BeWo cells [70]. Here, siRNA-mediated knockdown of BCL9L, β-catenin, or TCF4 in forskolin-treated BeWo cells all resulted in significant downregulation of forskolin-induced expression of GCM1 and syncytin-1 and syncytin-2 and fusion [70]. Similarly, TNF-α induced syncytin-1 expression in oral squamous carcinoma cells was dependent on Wnt/β-catenin signaling [46]. These data further substantiate the potential impact of β-catenin signaling in cell–cell fusion due to regulation of syncytins and other fusion-relevant protein expression. With regard to TNF-α-induced and MMP-9-dependent fusion of M13SV1-EGFP-Neo breast epithelial cells and human MDA-MB-435 cancer cells, the potential role of β-catenin signaling is not yet clear. E-Cadherin and MMP-9 are expressed by (TNF-α stimulated) M13SV1-EGFP-Neo breast epithelial cells but not MDA-MB-435 cells [57,58]. Thus, it might be assumed that MMP-9 mediated cleavage of E-Cadherin would result in release of β-catenin to the cytoplasm and nucleus concomitant with expression of fusion-relevant proteins only in M13SV1-EGFP-Neo breast epithelial cells. However, it cannot be ruled out that TNF-α-induced signal transduction pathways directly lead to β-catenin activation. This, for instance, has been demonstrated in glioma cells [71], and, more importantly, also in human breast epithelial cells [72].

Together, inflammation/inflammatory cytokines, such as TNF-α, can trigger cell–cell fusion, and some data point to involvement of β-catenin signaling. This could be either mechanistically (β-catenin degradation leads to actin reorganization) or transcriptionally (β-catenin-dependent gene expression), suggesting that β-catenin signaling might play a role in cell–cell fusion, e.g., by cellular conversion towards a pro-fusogenic state.

## 3. Effects of pH and Ions in Cell Fusion

Characteristically, many viral fusogens are initially in a non-fusogenic state. Only transition to an active, pro-fusogenic state after binding to the corresponding receptor by proteolytic cleavage or by pH-dependent irreversible conformational change leads to release in the fusogenic domain [4,5,10,73,74]. Interestingly, only certain viruses with class I, II, and III fusogens, such as influenza virus, HIV, Semliki Forest virus (SFV), dengue virus, and vesicular stomatitis virus (VSV), require low pH to be infectious [4,5,10,73,74]. Strictly speaking, there is no fusion with the plasma membrane of the host cell. Instead, viruses fuse with the membrane of endosomes because low pH values prevail in these vesicles. This rules out a direct role of low pH in cell–cell fusion. Accordingly, no pH dependency has been described for known human fusogens to date. However, a pH dependency was detected for two syncytin homologs in ruminants and lizards [75,76]. Indeed, syncytin-Rum1 and Mab-Env1 are fusogenic only at pH values of 5.0 and 4.0, respectively [75,76].

Data of Liang et al. proposed a pH-dependent mechanism of transmembrane member 16F (TMEM16F) activity [77]. Thereby, a low intracellular pH attenuated and a high intracellular pH potentiated TMEM16F channel and scramblase activity [77]. However, intracellular pH-dependent regulation of TMEM16F activity in normal cells and cancer cells remains elusive. If a change in intracellular pH for stimulation of Ca^2+^-activated phospholipid scramblases (Ca^2+^-PLS) is necessary for cell–cell fusion, this effect should be paralleled by a corresponding mechanism that is still obscure. It is known, however, that dysregulation of intracellular pH is one of the hallmarks of cancer cells [78]. Cellular pH is regulated by a variety of ion transporters and pumps, such as Na^+^-H^+^ exchangers, Na^+^-driven bicarbonate transporters, and H^+^/K^+^-ATPase proton pump [79,80]. However, it remains to be investigated by which processes/mechanisms these ion transporters and pumps could be specifically engaged to increase intracellular pH and enable Ca^2+^-PLS activation, whose activity is crucial for cell–cell fusion.

Interestingly, fusogenic properties have also been demonstrated for various positively charged ions, such as Ca^2+^ and Mg^2+^ [81]. For example, Ca^2+^ can induce fusion of PS vesicles or PS: phosphatidylcholine (PC) vesicles. This leads to attachment of positively charged Ca^2+^ ions to negatively charged PS or PS: PC membrane and destabilization of the structure. Accordingly, the total amount of positively charged Ca^2+^ ions as extracellular ionic stress affect phospholipid membranes by induction of aggregation and thereby potentially contributing to fusion [81].

**Figure 2:**
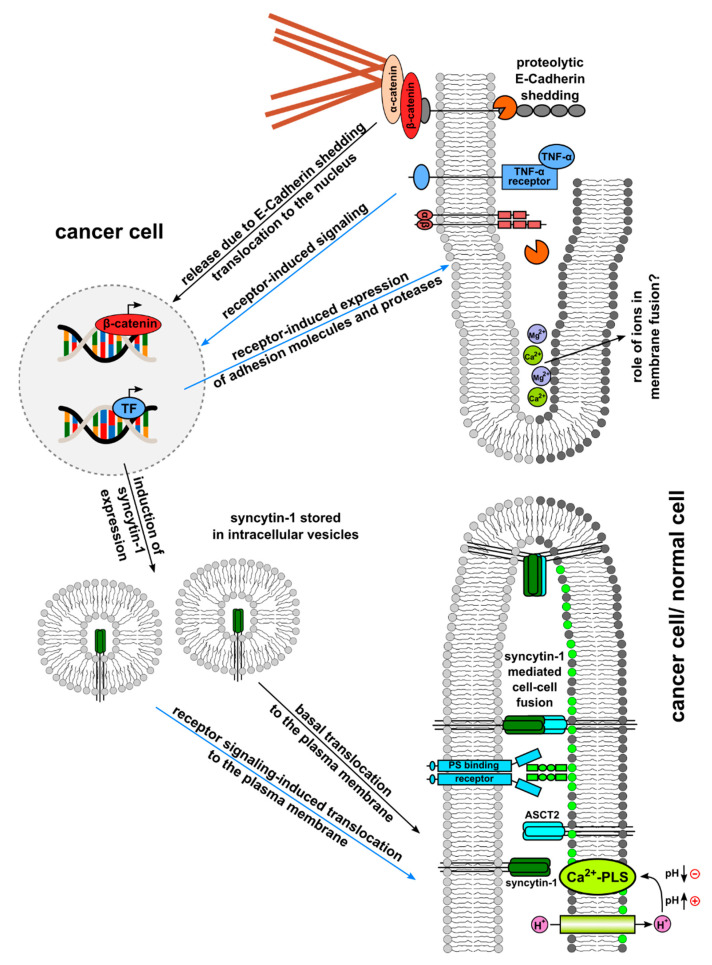
Cancer cell–cell fusion triggered by inflammation/inflammatory cytokines, ions, and pH. In this model based on a previous hypothetic scheme [1], it is assumed that cancer cells basally express syncytin-1 (and/or other fusogens) and a PS-binding receptor, which recognizes ASCT2 and PS, respectively. Fusogens such as syncytin-1 are stored predominantly in intracellular vesicles but could also translocate to the plasma membrane, allowing the cancer cell to enter a potential pro-fusogenic state. TNF-α-receptor-induced signaling could induce both fusogen (syncytin-1) expression and translocation to the plasma membrane. Similarly, TNF-α-receptor-induced signaling could induce expression of adhesion molecules and proteases. Adhesion molecules are mandatory for close cell–cell contact, which is a prerequisite for subsequent cell–cell fusion. Once cells have attached, adhesion molecules and other cell membrane proteins are cleaved by proteases. Proteolytic degradation of E-Cadherin was associated with release of β-catenin from the plasma membrane, nuclear translocation, and transcriptional activation of gene expression. Further extracellular triggers for (cancer) cell–cell fusion are positively charged ions, such as Ca^2+^, Mg^2+^, and H^+^. While Ca^2+^ and Mg^2+^ are also required for neutralization of negatively charged plasma membrane phospholipids, TMEM16F activity is modulated by intracellular H^+^ levels/pH values.

A summary of Section 2 and Section 3 is provided in Figure 2.

## 4. Virus-Mediated or -Associated Cancer Cell–Cell Fusion

### 4.1. Viruses as Bridgebuilders for Cell–Cell Merger

It is well-known that certain viruses, such as members of *Paramyxoviridae* (Sendai virus), *Retroviridae* (human immunodeficiency virus), *Coronaviridae* (SARS-CoV and SARS-CoV-2), *Poxviridae* (poxvirus) [82,83,84,85], and *Reoviridae* (Rota virus B) [86], exhibit fusogenic properties and can induce heterokaryon formation in various cell types. For instance, the Sendai virus was used for fusion of plasma cells and multiple myeloma cells to generate the first hybridomas [87].

Viruses can fuse cells by two different mechanisms [88]. In the first mechanism, a host cell is infected by a virus and virus-specific proteins, such as *env* elements in the cell membrane, are expressed [88]. Since these have fusogenic properties, fusion with other cells and formation of a heterokaryon can subsequently occur. Even sole expression of viral fusogens is sufficient, which is exploited, e.g., in therapy of oncolytic viruses [89,90,91]. In fact, oncolytic-virus-mediated fusion of cancer cells and formation of syncytia increases the effectiveness of therapy and targeted elimination of cancer cells [89,90,91]. To what extent viral membrane–cell membrane fusion can lead to accumulation of viral fusogens in the host cell membrane, including possible cell fusion events, is unknown. It might be assumed that this mechanism is rather unlikely. A certain concentration and possibly also an accumulation of fusogens in the plasma membrane would be necessary for interaction with corresponding receptors and fusion of the neighboring cell to occur. If the number of viral fusogens is too low, a dilution effect will quickly occur as they will spread quickly in the plasma membrane of the host cell.

In the second mechanism, it is believed that viruses do not infect the cells themselves but rather serve as a connective element that links two cells like a bridge, mediating cell fusion [88]. However, it is unclear whether such a mechanism exists and, if so, exactly how it would work. Ultimately, there must be a concerted action between cells and viruses here so that the viruses can fuse in parallel with two cells simultaneously.

With regard to cancer cell × normal cell fusions, however, it is unclear whether these can be mediated by viruses at all since viruses can only infect certain cells due to their tropism. For example, virus “A” can infect (cancer) cell “A” because it has the appropriate receptor “A” to which the virus can dock. (Cancer) cell “A” then expresses virus “A” *env* proteins so that it can fuse with other (cancer) cells “A”. Cell “B”, which does not have the receptor “A”, cannot be infected by virus “A”. Ergo, (cancer) cell “A” that expresses virus “A” *env* proteins cannot fuse with cell “B”.

However, since infection of (cancer) cell “A” with virus “A”, as described above, can lead to reactivation of HERV elements [92,93,94,95,96,97], it is conceivable that (cancer) cell “A” can then express HERV *env* proteins so that fusion with cell “B” can occur. A prerequisite for this is, of course, that cell “B” would have the appropriate receptors for HERV *env* proteins. Whether such a mechanism can take place at all is completely unclear and also very speculative.

### 4.2. Viral Infection Induced Reactivation of HERV env Elements

In addition to syncytin-1, expression of further human endogenous retroviral elements has been associated with cancer progression [98,99,100,101,102,103]. HERVs elements account for about 8% of the human genome [98,99,100,101,102,103]. The majority of them have become inactive due to mutations, translocation, deletions, or silencing [98,99,100,101,102,103,104]. Nonetheless, some of them are still transcribed and translated and have taken on other tasks, especially in early embryogenesis [98,99,100,101,102,103,104]. Interestingly, transcription and translation of HERV elements were also found in autoimmune diseases and cancer, whereby it is still not understood why and how HERV elements become reactivated.

#### 4.2.1. HERV-K (HML-2) and Cancer

Of all known HERVs, the HERV K family, particularly the HML-2 subgroup, is the youngest and most transcriptionally active [98,99,100,101,102,103]. More than 1,000 HERV-K (HML-2) loci are found in the human genome [103]. Many of these loci possess complete open reading frames (ORFs) with coding capability, expression of HML-2 transcripts and proteins, and even production of retrovirus-like particles [98,99,100,101,102,103]. It is well-known that the retroviral envelope (*env*) protein is important for membrane fusion of retroviruses [98,99,100,101,102,103]. Interestingly, HERV-K (HML-2) *env* expression was found in several cancers, such as breast cancer, melanoma, hepatocellular carcinoma, pancreatic cancer, Karposi’s sarcoma, and leukemia, and were further associated with disease progression [98,99,100,101,102,103]. For instance, elevated *env* transcription and protein levels were correlated to increased disease/TNM staging and lymph node positivity [105,106,107]. Similarly, elevated *env* levels were associated with markedly decreased overall/disease-free survival in breast cancer, melanoma, and hepatocellular carcinoma [105,106,107]. Likewise, in vitro and in vivo studies revealed that proliferation, invasion, migration, tumor growth, and metastasis were markedly enhanced in HERV-K (HML-2)-*env*-expressing breast cancer, pancreatic cancer, and Karposi’s sarcoma cells due to more active Ras/Raf/MAPK signaling [94,108,109,110]. Whether HERV-K (HML-2) *env* protein expression might also promote cancer progression by cell–cell fusion is not clear. To date, only one study has demonstrated HERV-K (HML-2)-*env*-element-encoded fusogenic activity in melanoma cells [111]. However, the prospective fusogenecity of HERV-K (HML-2) *env* in melanoma cell fusion was only investigated in RNAi and blocking antibody studies [111]. Both approaches resulted in a dramatic reduction in melanoma hybridization, which substantiates the assumption that HERV-K (HML-2) *env* may promote cell–cell fusion [111]. However, appropriate overexpression studies were not conducted. Hence, non-fusogenic cells should become fusogenic when expressing HERV-K (HML-2) *env*, which would support the hypothesis that HERV-K (HML-2) *env* still exhibits fusogenic properties.

#### 4.2.2. Reactivation of HERV Elements Due to Viral Infections

It is well-known that infection with exogenous viruses, such as HIV, hepatitis B virus (HBV), Epstein–Barr virus (EBV), Influenza A, Karposi’s sarcoma herpes virus (KSHV), and SARS-CoV-2, can induce significant HERV transactivation concomitant with elevated HERV element expression levels, including the *env* protein [92,93,94,95,96,97,102,112]. For instance, HERV-K (HML-2) *env* expression was induced in KSVH-infected cells through LANA-enhanced ERK activity and vFLIP-induced NF-κB activity [94]. Syncytin-1 expression was induced through Hepatitis B virus X protein (HBx)-engaged NF-κB activation in HepG2 hepatocellular carcinoma cells [93]. This is in agreement with the prospective correlation of elevated syncytin-1 expression levels and disease progression in hepatocellular carcinoma [113]. Similarly, expression of syncytin-1 could be induced by EBV, HIV, and SARS-CoV-2 infections [95,96,97]. EBV- and HIV-associated/caused diseases, such as multiple sclerosis (EBV) and neuroinflammation (HIV), might be related to EBV/HIV-induced syncytin-1 expression [95,96,97]. However, the impact of syncytin-1 up-regulation in the pathogenesis of SARS-CoV-2 is not yet clear [95,96,97].

Interestingly, even retroviral transduction of cells, which is routinely used to generate cell lines expressing exogenous non-viral genes, sufficiently induced syncytin-1 expression in transduced PC3 prostate cancer cells [114]. Moreover, exosomes derived from retrovirally transduced cells carried syncytin-1 and were able to transduce target cells via horizontal gene transfer. This supports the assumption that endogenous syncytin-1 mediates delivery of extracellular vesicle cargo into target cells through fusion [114]. Indeed, syncytin-1 was found in the membranes of placenta-derived exosomes, which was important for exosome-mediated intercellular communication [115,116]. However, the data of Uygur and colleagues showed that the sole presence of syncytin-1 in the membrane of exosomes was not sufficient to mediate gene transfer [114]. This was further dependent on MLV-GAG, which was encoded by the retroviral construct used for transduction [114]. The authors hypothesized that MLV-GAG promoted syncytin-1-mediated fusion, possibly by raising local concentration of syncytin-1 [114]. Briefly, these findings indicate that retroviral transduction of cells likely induces HERV element expression in target cells, which might lead to the origin of exosomes sharing some similarities with retroviruses. In this regard, it would be interesting to investigate whether other retroviruses, such as HIV, or reactivation of HERV elements may also lead to generation of syncytin-1 or other *env*-proteins-positive retroviral-like exosomes.

In summary, an increasing body of evidence reveals a crucial role of reactivated HERV elements, especially syncytin-1 and HERV-K (HML-2), in cancer progression [98,99,100,101,102,103]. However, the mechanism of reactivation of HERV elements is still scarcely understood. Hence, it remains unknown whether aberrant expression of HERV elements might be a direct cause of the disease or solely plays a role in disease progression. A possible link between cell–cell fusion and cancer progression has so far only been demonstrated for HERV *env* element syncytin-1. In addition to its fusogenic properties, increased syncytin-1 expression also has an impact on cancer cell proliferation [44,45,46,48,50,117,118]. In contrast, the prospective impact of other HERV *env* elements in cell–cell fusion in cancer remains to be elucidated. Data of Huang et al. demonstrated that the HERV-K (HML-2) *env* element principally exhibited fusogenic properties [111], but this has only been conducted in melanoma cell lines so far. Thus, additional studies with further cancer cell lines of different tumor entities should be performed first to validate the prospective fusogenic properties of HERV-K (HML-2) *env* in cancer. Exogenous viruses have been further identified as reactivators of HERV element expression in various cell types [92,93,94,95,96,97,102,112]. In this regard, it would be interesting to investigate whether infection of cancer cells with exogenous viruses would also have an impact on the tumor cells’ HERV element expression pattern. Moreover, it would be worthwhile to speculate whether reactivation of a specific HERV subtype, such as HERV-K (HML-2), might also have an impact on the gene expression of other HERV subtypes/elements, such as syncytin-1.

## 5. Role of Hypoxia in Cell Fusion

A characteristic feature of advanced cancers is a hypoxic TME [119,120,121,122]. Of particular importance is activation of transcription factor hypoxia-inducible factors-1α (HIF-1α), which leads to expression of numerous hypoxia-associated target genes [119,120,121,122]. The specific role of these genes/proteins in cancer cell–cell fusion remains to be investigated. This further includes the question of which cell types in the hypoxic and inflammatory TME are converted to a pro-fusogenic state by hypoxia. The TME consists of various fusogenic cell populations, such as tumor cells, MSC, macrophages, stem cells, and fibroblasts [119,120,121,122]. All of them respond to hypoxic conditions with altered gene expression profiles and phenotypes [119,120,121,122]. Indeed, several studies suggest an impact of hypoxia on cell–cell fusion of various cell types. These include myoblasts and MSC [123], urine-derived stem cells [124], human hematopoietic progenitors and murine cardiomyocytes [65], ovarian cancer cells [125], breast cancer cells [60], and OSCCs and endothelial cells [126]. In contrast, previous in vitro studies demonstrated that both cell–cell fusion and differentiation of cytotrophoblasts and BeWo cells were impaired by hypoxia due to altered syncytin-1 expression and function [127,128]. This might be an explanation for a reduced syncytia formation in placentas of certain pathological pregnancies [127], indicating a placenta-specific hypoxia-reduced fusion frequency.

Nevertheless, all studies reporting an association between hypoxia and cell–cell fusion provide only very limited explanation for a potential molecular link on how hypoxia might be connected with cell fusion. In this context, fusogens such as syncytin-1 were not investigated [60,65,124,125,126]. Solely the study by Archaka et al. showed that hypoxia increased expression of the transcription factors myoD and myoG [123], which induced expression of fusogens myomaker and myomerger [64]. In association with cytokines, hypoxia and IL-6 or TNF-α increased the fusion of human peripheral blood CD34^+^ cells and murine cardiomyocytes through up-regulation of α_4_β_1_-integrin (VLA-4) and VCAM-1 [65]. Inhibition of this interaction using blocking antibodies markedly impaired cell–cell fusion [65]. Hu and colleagues demonstrated that fusion of hypoxia preconditioned human urine-derived stem cells with murine hepatocytes in a chronic injured mouse model was dependent on up-regulation of CXCR4 signaling [124]. Our own data revealed that hypoxia and TNF-α-mediated fusion of human breast cancer cells and human breast epithelial cells were attributed to induction of MMP9 expression in human breast epithelial cells [58,60]. These findings substantiate the importance of chemokine receptor signaling, adhesion molecules, and tight cell–cell contact as prerequisites for cell–cell fusion. Nonetheless, it remains unclear which proteins ultimately facilitated plasma membrane merger in these studies. Huang and colleagues demonstrated that hypoxia promoted spontaneous cell–cell fusion between OSCCs and human immortalized oral epithelial cells partly via epithelial-to-mesenchymal transition (EMT) in epithelial cells [126]. Treatment of epithelial cells with the EMT blocker DAPT significantly reduced the fusion rate of the cells [126]. Induction of EMT and DAPT-blocked EMT was validated by Western blot of known EMT markers, including N-Cadherin, E-Cadherin, Vimentin, and Slug [126]. Unfortunately, no further protein expression studies have been performed [126], so the impact of EMT on the fusion of these cells remains ambiguous.

Although an effect of hypoxia on cell–cell fusion is evident, it remains unclear which cell type is primarily converted to a pro-fusogenic state and what exactly happens at the molecular level.

## 6. Exosomes/EVs in Cell Fusion

Exosomes represent distinct kinds of EVs contributing to cell fusion. The size, heterogeneity, and complex composition of exosomes/EVs creates challenges for use of conventional analytical tools and strategies. Thus, the approximately 20 to 200 nm vesicles display typical exosome marker proteins, including surface glycoproteins of the tetraspanin transmembrane-4 family, such as CD9, CD63, and CD81 [129]. Exosomes play an important role during intercellular communication, e.g., between cancer cells and MSC and potentially during cell–cell fusion [130]. The presence of PS, particularly in tumor-derived exosomes, represents a prerequisite for fusogenic properties [131]. Exosomes develop from multivesicular bodies of endolysosomal pathway origin followed by budding of late endosomes that subsequently fuse with the plasma membrane to release their content [132,133]. The resulting exosomes contain a double membrane and encapsulate different proteins, DNA fragments, and RNAs. That is, a large variety of RNA components are associated with exosomes, such as mRNAs, circRNAs, tRNAs, long non-coding RNAs, and regulatory microRNAs (miRNAs). The biological content of exosomes differs among the originating cell type and/or the tissue. For example, bone-marrow-derived MSC as compared to adipose tissue-derived MSC contain distinct sets of tRNAs and miRNAs [134].

Released exosomes are shuttled in vivo via biological fluids, such as blood, interstitial fluid, urine, saliva, or cerebrospinal fluid [132,135], to alter the functionality of recipient cells, including cancer cells [136,137,138,139]. Taking clinical advantage of this process, e.g., MSC-derived exosomes can be used to cure diseases [140] or can be loaded with therapeutics to target tumors [141,142]. Uptake of exosomes and EVs by appropriate target cells was suggested by a three-step mechanism: (i) targeting of the acceptor cell by EVs; (ii) entry point and internalization of EVs into the acceptor cells; (iii) delivery of EV content to the acceptor cell [143]. These exosome internalization steps involve clathrin-dependent and -independent pathways, macropinocytosis, phagocytosis, lipid-raft-mediated internalization, receptor-mediated endocytosis, and EV fusion with the target cell plasma membrane [136,144].

Although direct proof remains to be elucidated, previous work suggested that exosomes/EVs could directly mediate cell–cell fusion, e.g., by acting as a linker to bridge two individual cells. Previous work has demonstrated that exosomes released by trophoblasts contain fusogens, such as syncytin-1, in their membrane. This seems to be important for exosome × target cell membrane merger and delivery of extracellular vesicle cargo into target cells [145]. However, it remains to be elucidated whether syncytin-1-positive exosomes might be capable to bridge two cells and thus may facilitate cell–cell merger [145,146].

**Figure 3:**
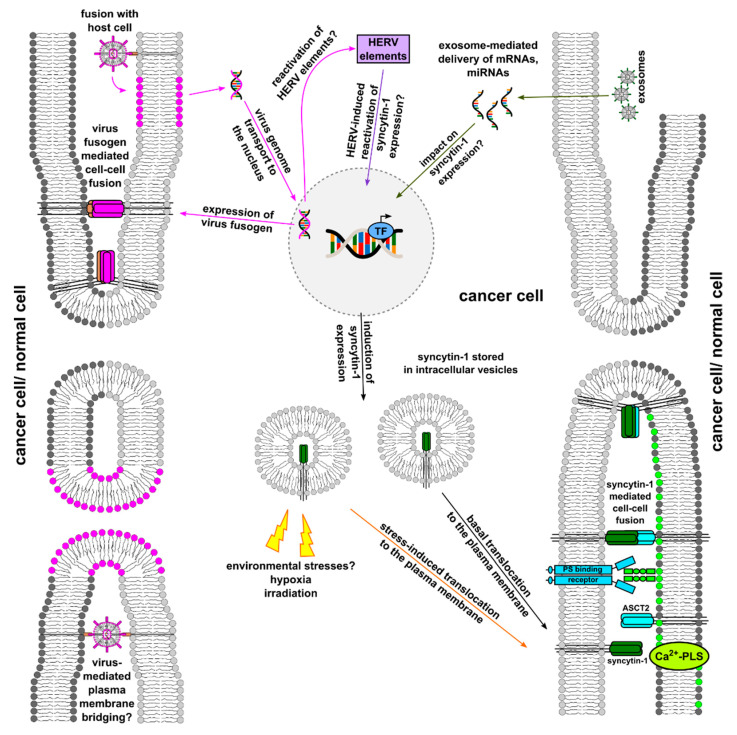
Cancer cell–cell fusion triggered by environmental stress, viruses, and exosomes. In accordance to Figure 2, fusogens and their cognate receptors, as well as PS and PS-binding proteins, are indispensable for cancer cell–cell fusion. Induction of fusogen (syncytin-1) expression could be putatively triggered by mRNAs and miRNAs delivered by exosomes. The role of viruses in cancer cell–cell fusion is less clear. Although still unclear, cell-surface-associated viruses may directly act as bridging elements for two individual cells. Moreover, virus infection and subsequent expression of viral fusogens represent another mechanism that might cause membrane merger of two (or more) individual cells. A potential re-activation of syncytin-1 expression in cancer cells by HERV elements and a fusion-related role of other virus-derived elements upon infection remain less clear. Extracellular stress, such as hypoxia and/or irradiation, might trigger translocation of fusogens (syncytin-1) from intracellular vesicles towards the plasma membrane.

A summary of Section 4, Section 5 and Section 6 is provided in Figure 3.

Fusion of cancer cells suggests that this process is controlled by intrinsic and extracellular factors. In the present review, we have focused on those extracellular conditions that act from outside the cells to induce/mediate cell–cell fusion, such as inflammation/inflammatory cytokines, viruses, pH, ion concentration, hypoxia, and EVs/exosomes. Pro-fusogenic effects have been reported predominantly by inflammation/inflammatory cytokines and hypoxia [46,52,53,54,55,60,65,123,124,125,126]. However, so far, it is completely unclear what this pro-fusogenic effect looks like at the molecular and cellular level. Do hypoxia and inflammation/inflammatory cytokines induce pro-fusogenic proteins/factors in the target cells or increased translocation of fusogens into the cell membrane, or both? This needs to be clarified in further studies. The relationship between inflammation and hypoxia and cell–cell fusion is also interesting for this reason since the TME is both hypoxic and chronically inflamed [119,120,121,122,147,148,149].

To what extent viruses, a low pH, or exosomes have an influence on the fusogenecity of cells or even contribute to fusion of two (or more) (cancer) cells also remains unclear. It appears conceivable that viruses and exosomes can induce fusion of two or more cells as bridging elements, but it remains to be verified in vivo. This also applies to possible reactivation of HERV *env* elements with fusogenic properties by extracellular factors and their potential contribution to cancer cell–cell fusion.

## 7. Conclusions

The process of cell–cell fusion in the tumor context remains poorly understood. It is now scientific consensus that cell–cell fusion events occur in tumors, even in human cancers [150,151,152,153,154,155,156,157,158,159]. Nonetheless, the questions remain not only by which proteins/factors/conditions this process is mediated but also how it is regulated in general. Likewise, it remains unclear why cancer cells and other cells can fuse with each other and whether this is a random or directed process. Cancer cell fusion increases tumor plasticity via alternative developments, including enhanced metastatic spreading [17], reduced tumorigenicity [22], or tumor dormancy [28]. These diverse outcomes suggest different rather than common pathways or conserved mechanisms that are responsible for both initiation and progression of cancer cell fusion with subsequent PHSP. The previously summarized aneuploidy of individual cell lines from a variety of different tumor entities substantiates potential cancer cell fusion and corresponding tumor heterogeneity [61]. Moreover, underlying inflammatory processes within the TME may similarly differ broadly across different organs and tumor sites.

Although the process of cell–cell fusion has been further elucidated in recent years, much remains unclear. This is primarily true for cancer cell fusion in tumor tissues controlled by intrinsic and extrinsic factors/mechanisms. In this context, extrinsic factors/mechanisms represent potential targets/target structures that could be exploited therapeutically. For example, pro-inflammatory cytokines, such as the presence of TNF-alpha, could be modulated to decrease the cell–cell fusion rate in the TME. Similarly, specific protease inhibitors could be used to alter proteolytic degradation of cell–cell contacts and thereby reduce cell–cell fusion. Moreover, exosomes and their potential role in cell–cell fusion also require further investigation. It is known that MSC-derived exosomes have anti-inflammatory properties and confer immunomodulatory effects [140,160]. The TME resembles chronically inflamed tissue and thus represents a potentially pro-fusogenic environment, which could favor fusion of cancer cells with other cells. Due to their anti-inflammatory and immunomodulatory properties, exosomes could interfere with this process.

Together, cell–cell fusion of cancer cells can be promoted by extracellular stimuli, whereas these extracellular factors may not directly mediate the merger of two (or more) cancer cells. Instead, extracellular events support appropriate intrinsic factors that subsequently mediate cell–cell fusion. Orchestrating intrinsic components confer signals for a pro-fusogenic state according to our fusion models (Figure 1, Figure 2 and Figure 3). The involved factors together with structural and metabolic changes include, among others, G-actin to F-actin cytoskeletal reorganization, phosphatidylserine accumulation in the plasma membrane, and expression of fusogens (e.g., syncytin and corresponding receptors) to promote a pro-fusogenic state. Consequently, extracellular conditions provide an important prerequisite to relay outside-in signaling for further supporting and promoting the complex process of (cancer) cell fusion.

## Data Availability

Not applicable.

## Abbreviations

ASCT2	alanine, serine, cysteine transporter 2 (syncytin-1 receptor)
BMDCs	bone-marrow-derived cells
EMT	epithelial–mesenchymal transition
Ca^2+^-PLS	Ca^2+^-activated phospholipid scramblases
EBV	Epstein–Barr virus
EMT	epithelial-to-mesenchymal transition
EVs	extracellular vesicles
GCM1	glial cell missing-2
HBV	hepatitis B virus
HERV	human endogenous retroviral
HERV-FRD	human endogenous retrovirus-type FRD (syncytin-2)
HER2-W	human endogenous retrovirus-type W (syncytin-1)
HUVECs	human umbilical vein endothelial cells
HIF-1α	hypoxia-inducible factors-1α
HIV	human immunodeficiency virus
IL-6	interleukin-6
KSHV	Karposi’s sarcoma herpes virus
MMP-9	matrix metalloproteinase-9
MSC	mesenchymal stroma-/stem-like cells
OSCCs	oral squamous carcinoma cells
PHSP	post-hybrid selection process
PS	phosphatidylserine
PC	phosphatidylcholine
SARS-CoV-2	severe acute respiratory syndrome coronavirus-2
SCC-9	squamous cell carcinoma cells 9
SFV	Semliki Forest virus
TCF4	T-cell factor 4
TME	tumor microenvironment
TMEM16F	transmembrane member 16F
TNF-α	tumor necrosis factor-α
VCAM-1	vascular cell adhesion molecule-1
VLA-4	very late antigen-4
VSV	vesicular stomatitis virus
